# The ectodomain of cadherin-11 binds to erbB2 and stimulates Akt phosphorylation to promote cranial neural crest cell migration

**DOI:** 10.1371/journal.pone.0188963

**Published:** 2017-11-30

**Authors:** Ketan Mathavan, Vikram Khedgikar, Vanessa Bartolo, Dominique Alfandari

**Affiliations:** Department of Veterinary and Animal Sciences, University of Massachusetts Amherst, Amherst, Massachusetts, United States of America; University of South Alabama Mitchell Cancer Institute, UNITED STATES

## Abstract

During development, a multi-potent group of cells known as the cranial neural crest (CNC) migrate to form craniofacial structures. Proper migration of these cells requires proteolysis of cell adhesion molecules, such as cadherins. In *Xenopus laevis*, preventing extracellular cleavage of cadherin-11 impairs CNC migration. However, overexpression of the soluble cleavage product (EC1-3) is capable of rescuing this phenotype. The mechanism by which EC1-3 promotes CNC migration has not been investigated until now. Here we show that EC1-3 stimulates phosphorylation of Akt, a target of PI3K, in *X*.*laevis* CNC. Through immunoprecipitation experiments, we determined that EC1-3 interacts with all ErbB receptors, PDGFRα, and FGFR1. Of these receptors, only ErbB2 was able to produce an increase in Akt phosphorylation upon treatment with a recombinant EC1-3. This increase was abrogated by mubritinib, an inhibitor of ErbB2. We were able to recapitulate this decrease in Akt phosphorylation *in vivo* by knocking down ErbB2 in CNC cells. Knockdown of the receptor also significantly reduced CNC migration *in vivo*. We confirmed the importance of ErbB2 and ErbB receptor signaling in CNC migration using mubritinib and canertinib, respectively. Mubritinib and the PI3K inhibitor LY294002 significantly decreased cell migration while canertinib nearly prevented it altogether. These data show that ErbB2 and Akt are important for CNC migration and implicate other ErbB receptors and Akt-independent signaling pathways. Our findings provide the first example of a functional interaction between the extracellular domain of a type II classical cadherin and growth factor receptors.

## Introduction

Cranial neural crest (CNC) cells are a highly plastic population of cells that are responsible for craniofacial formation during embryogenesis. Originating at the border of the neural and non-neural ectoderm, CNC cells migrate ventrally into the developing face and differentiate into numerous cell types, including bone and cartilage [1]. Improper development and migration of the CNC can result in clefting of the palate and other malformations of the face [2]. Vital to these events are the precise regulation and processing of cadherins [3–8].

Cadherins belong to a superfamily of cell surface proteins involved in cell-cell adhesion and migration. In *Xenopus*, cadherin-11, N-cadherin, and E-cadherin are required for CNC migration [8–10]. These classical cadherins are composed of an adhesive extracellular domain, a transmembrane domain and a cytoplasmic tail. The intracellular domain can bind to the actin cytoskeleton through a multi protein complex that includes of α– and β-catenin, effectively providing a connection for cells between the plasma membrane and the cytoskeleton [11]. In *Xenopus*, replacement of cadherin-11 with a mutant that is unable to bind to β-catenin prevents *in vivo* cell migration and induces blebbing of CNC cell membranes *in vitro* [9]. Classical cadherins also have a region for binding p120 catenin on their cytoplasmic tail [12]. In pre-migratory neural crest cells, association of p120 with E-cadherin is necessary to suppress contact inhibition of locomotion and thereby prevent precocious CNC migration [13].

The extracellular domains of classical cadherins are best known for their role in cell adhesion. This region is composed of five beta-folded cadherin (EC) repeats and allows these cadherins to form lateral (*cis*) homodimers or connect adjacent cells through *trans* homodimers. Cell-cell adhesion is facilitated through the distal most EC domain (EC1) of classical cadherins by inserting conserved tryptophan residues into a hydrophobic pocket belonging to an EC1 domain of an opposing cadherin [14,15]. Classical cadherins are subdivided into two groups depending on the way they form *trans* interactions. Type 1 cadherins, such as E-cadherin, utilize a single tryptophan and a hydrophobic pocket defined by a conserved histidine-alanine-valine (HAV) motif [14]. On the other hand, cadherin-11 and other type II cadherins require two tryptophan residues for binding, and often have a QAV sequence in place of the HAV motif [15,16]. Mutation of these conserved residues eliminates the adhesive activity of classical cadherins [15,17]. Substitution of E-cadherin or cadherin-11 with mutant forms lacking their homophilic site inhibits proper CNC migration in *Xenopus* [3,7,8].

At the cell surface, matrix metalloproteases (MMPs) and ‘a disintegrin and metalloproteinases’ (ADAMs) shed cadherin ectodomains from their membrane-bound halves and subsequently allow gamma-secretase to cleave cadherin intracellular domains [18,19]. In chick, cleavage of N-cadherin or cadherin-6B ectodomains by ADAM10 or ADAM19 precedes the release of their cytoplasmic domains, which translocate into the nucleus to regulate gene expression [5,20]. The release of cadherin ectodomains has been shown to influence the migratory behavior of cells [21–23]. For example, treatment of Madin-Darby canine kidney (MDCK.ts-*src*C12) cells with soluble E-cadherin fragments (sEcad), generated by MMP2 or MMP7, can promote invasion of these cells into collagen type I matrices [21]. In CNC cells, ADAM13 cleaves cadherin-11 between its third and fourth cadherin domain [23]. We have shown that this fragment, EC1-3, can rescue CNC migration in embryos depleted of ADAM13 as well as those overexpressing cadherin-11 [23]. While EC1-3 retains its ability to form homophilic interactions, this activity is not needed to promote migration in the CNC [24]. Although cadherin ectodomains were first implicated in pro-migratory behavior several years ago, it wasn’t until recently that the mechanisms behind this phenomenon were investigated.

Studies of E-cadherin have revealed that cadherin fragments can bind to epidermal growth factor receptors (ErbBs) and signal through downstream pathways [25–28]. Initial experiments with sEcad demonstrated that it could mediate ErbB2-ErbB3 heterodimerization in MCF-7 breast cancer cells [25]. This interaction activates the two receptors and is thought to be responsible for the increased levels of phosphorylated mitogen-activated protein kinase (MAPK) observed in the breast cancer cells [25]. The signaling potential of sEcad was further expanded in recent studies on squamous cell carcinomas and breast cancer cells [27,28]. These groups showed that sEcad could associate with epidermal as well as insulin growth factor receptors to stimulate MAPK and phosphoinositol 3-kinase (PI3K)/Akt/mammalian target of rapamycin (mTOR) pathways. Furthermore, inhibition of EGF receptors, MAPK or PI3K decreased the pro-migratory effects of sEcad. The importance of PI3K signaling in *X*.*laeivs* CNC migration was recently reported and appears to facilitate contact inhibition of locomotion by promoting expression of N-cadherin [29].

In this study, we use embryological and cell culture experiments to elucidate the mechanisms by which the shed cadherin-11 ectodomain promotes CNC migration. We show that EC1-3 stimulates Akt phosphorylation in Hek293T and CNC cells. Although we show that EC1-3 can bind to several growth factor receptors, it is only through ErbB2 that EC1-3 activates Akt in Hek293T cells. Knocking down the receptor in *Xenopus laevis* embryos decreased Akt phosphorylation in CNC cells and reduced their migration *in vivo*. The ErbB2 inhibitor mubritinib also significantly reduced CNC cell migration, confirming the importance of ErbB2 in CNC motility. For the first time we demonstrate the potential of a type II, classical cadherin ectodomain to bind ErbB receptors and stimulate PI3K/Akt signaling, which we show are both critical for CNC migration.

## Material and methods

The use of Xenopus embryo was approved under the animal protocol number 2015–0029 by the IACUC of the University of Massachusetts Amherst.

### DNA constructs

All ErbB receptor constructs were generously donated by Dr. Chenbei Chang (University of Alabama, Birmingham) [30]. EGFR/ErbB1 and ErbB4 were human sequences while ErbB3 was from rat. The sequences for wild type and dominant negative (DN) ErbB2 construct were from *Xenopus laevis*. A rat sequence of ErbB2 was used as a rescue construct for morpholino experiments. Restriction-free cloning was used to insert a flag tag downstream of ErbB2 and DN-ErbB2. FGFR1 and PDGFRα were cloned from *X*.*laevis* cDNA into pCS2+ by PCR and in frame with a flag tag. Myc-tagged EC1-3 was previously described [23]. Non-adhesive EC1-3-myc (naEC1-3-myc) was also previously described [24]. In-Fusion HD cloning (Clontech) was used to insert a tandem of six myc tags downstream of EC1. Dr. Michael Klymkowsky (University of Colorado Boulder) generously donated the UGP- and GFP-myc constructs.

### Protein purification

Amino acids 54–385 of cadherin-11 from *Xenopus laevis*, followed by six histidines, were cloned into pET28 to produce bacEC. The construct was transformed into BL21(DE3) *E*.*coli* and 500 mL were grown to an OD_600_ equal to 0.6. The culture was then induced for 3 hours with 1 mM IPTG. Bacterial pellets were resuspended in a denaturing buffer composed of 10 mM Tris, 100 mM sodium phosphate, 8 M urea, pH 8.0. Lysates were cleared by centrifugation at 10,000g for 30 min at 4°C. Ni-NTA beads (Qiagen) were then added to lysates, inverted overnight at 4°C and washed with a denaturing buffer similar to the one above but adjusted to pH 6.3. Captured protein was gradually renatured on a gravity column using a calcium-magnesium free (CMF) buffer composed of 88 mM sodium chloride, 1 mM potassium chloride, 2.4 mM sodium bicarbonate, 15 mM Hepes, pH 7.6. Protein was then eluted in 400 mM imidazole in CMF and dialyzed in phosphate-buffer saline.

### Cell culture experiments

Hek293T cells (ATCC) were grown in RPMI-1640 media supplemented with 50IU/0.05 mg/mL of penicillin/streptomycin (MP Biomedicals), 2 mM L-glutamine (Hyclone), 1 mM sodium pyruvate (Gibco), and 10% fetal bovine serum (Atlanta Biologicals). Cells were transfected using X-tremeGENE HP (Roche) at a 2:1 ratio of transfection reagent to DNA. Cell media was exchanged after 24 hours for immunoprecipitation experiments in order to maintain healthy cells. After an additional 24 hours, cells were lysed in 1xMBS, 1% Triton X-100, 5 mM EDTA, and 1x Halt Protease and Phosphatase Inhibitor Cocktail (Thermo Scientific; #78440). Immunoprecipitations were performed with Protein A/G Plus-Agarose (Pierce; #20423), anti-HA (abm; #G036) and anti-FLAG M2 (Sigma-Aldrich; #F3165) antibodies. Proteins were detected using anti-myc 9E10 (DSHB) and anti-PI3K p85 (Cell Signaling; #4257). For detection of phospho-Akt (S473; Cell Signaling; 4051), cells were starved after transfection for 18–20 hours in supplemented RPMI-1640 media without serum. Cells were then treated with 10 ng/mL of bacEC for indicated lengths of time. Cells were directly extracted in reducing Laemmli buffer. For inhibition experiments, cells were treated with 600 nM mubritinib or 0.3% DMSO (v/v) for one hour prior to treatment with bacEC. GFP-myc was co-transfected with receptors and used to normalize phospho-Akt levels. Quantification of western blots was performed in Adobe Photoshop using a combination of the Rectangular Marquee Tool and Measurement Log. X-rays at the lowest exposure, to avoid saturation, were scanned as TIFFs (600dpi). Integrated densities were recorded for proteins of interest and identical areas without signal for background correction. For some experiments, western blots were imaged directly on a G-Box (Syngene) and quantified using the Syngene software. Both methods gave similar results but the X-Ray were more sensitive and were therefore used for experiment using embryos or explants.

### Embryo manipulations

For overexpression experiments embryos were injected into a single blastomere at the 2-cell stage with 200 pg of UGP mRNA alone or combined with 800 pg of EC1-3-myc mRNA. Embryos were then sorted at neurula stages based on fluorescence of UGP and ten corresponding CNC explants were dissected and lysed as above for western blotting. In all assays, explants were dissected in 1xMBS with 10 μg/mL gentamycin and placed in Danilchik media [31] for 10 minutes before further processing. This was done to eliminate unhealthy cells from downstream steps. The following antibodies were used for protein detection: anti-phospho-Akt (S473; Cell Signaling; 4051), anti-Akt (Cell Signaling; 4691), anti-ACTIVE MAPK (Roche; V803), anti-GAPDH (EMD Millipore; MAB374). All injected mRNA was transcribed *in vitro* as previously described [32].

Experiments with bacEC were performed on CNC dissected from non-injected embryos. Explants were treated with 10 ng/mL of bacEC in Danilchik media supplemented with 1 mg/ml BSA and 10 μg/mL gentamycin for 5 minutes before being transferred to a new tube, lysed and analyzed via western blotting. Immunodepletion of bacEC from treatment solution was performed using Protein A/G Plus-Agarose (Pierce; #20423) conjugated to an anti-EC1-3 mouse monoclonal, 4F12. Conjugation and immunodepletion were performed for 30 minutes and 2 hours at room temperature, respectively. The monoclonal 4F12 was generated by immunizing BALB/c mice (Jackson Laboratories) with bacEC. Hybridomas were obtained by standard technique and screened by ELISA on bacEC. Positive hybridomas were tested for reactivity on EC1-3 expressed in Hek293T cells to eliminate clones directed against the purification tag.

Knockdown of ErbB2 was accomplished using a translation-blocking morpholino (MO ErbB2). The sequence of the morpholino CAGCTCCATCATC-TACTCCATGTCC was complementary to 13 bases of the 5’UTR and 12 bases of the open reading frame of *Xenopus laevis* ErbB2. Knockdown was confirmed by injecting single-cell embryos with 800 pg of DN-ErbB2-Flag mRNA with or without 25 ng of the morpholino. Embryos were grown for one day at 14°C and lysed for western blotting. To check phosphorylation of Akt in CNC, embryos were injected with 200 pg of RFP mRNA with or without 3.1 ng of MO ErbB2 into the right two animal pole cells at the 8-cell stage. Embryos were grown at 14°C for two days until they reached stage 17–18. After confirming RFP fluorescence on the right side of embryos, corresponding CNCs were dissected, allowed to heal and lysed as described above. Induction of CNC was assayed by taking the RFP and MO ErbB2-injected embryos above and fixing them at stage 18. Whole mount *in situ* hybridization using *Twist* and *Sox10* probes was then performed to demarcate the CNC as previously described [33]. Photographs were taken on a Zeiss SteREO.LumarV12 using an AxioCam HRc.

### Migration assays

Inhibitors were used at the following concentrations to disrupt CNC migration *in vivo*: 30 μM of LY294002 (IC_50_ = 1.4μM; Millipore-Calbiochem, #440202), 40 μM of rapamycin (IC_50_ = 0.1nM; Millipore-Calbiochem, #553211), 40 μM mubritinib (IC_50_ = 6nM; Selleckchem; #S2216), and 25 μM canertinib (EGFR IC_50_ = 1.5nM; ErbB2 IC_50_ = 9nM; Selleckchem; #S1019). DMSO was added as a control to a final concentration of 0.15–1.25% (v/v). Embryos were treated at neurula stages (st.16-17) to avoid disruption of gastrulation and neural crest specification. Vitelline membranes were removed prior to treatment to facilitate access of inhibitors to CNC cells. After being grown at 14°C overnight, embryos were collected at early tailbud stages (st.25-26) and fixed in MEMFA (100 mM MOPS, 2 mM EGTA, 1 mM magnesium sulfate, 3.7% formaldehyde). For morpholino experiments, embryos were injected with combinations of 200 pg of RFP mRNA, 1.6 ng of MO ErbB2, and 300 pg of rat ErbB2 mRNA into the two right animal pole cells at the 8-cell stage. Embryos were grown at 14°C for three days and processed as above. Embryos were then labeled for CNC markers and photographed as above. The Line tool in Fiji [34] was used to measure the length of each CNC stream as well as the head height. We calculated the percent each CNC stream traveled by dividing each segment length by the head height and multiplying by one hundred.

*Ex vivo* assays were performed by dissecting CNC as detailed above and placing one explant per well in a polystyrene 96-well plate coated with 10 μg/mL fibronectin (Sigma-Aldrich; #F1141) as previously reported [35]. Explants were placed at 18°C for at least one hour before 0.5% DMSO (v/v), 6 μM mubritinib, 10 μM canertinib, or 20 μM LY294002 were added. This ensured explants attached to the fibronectin substrate prior to treatment and imaging. A 10 ng/mL concentration of bacEC was used for persistence experiments. Explants were kept at 18°C and photographed every 30 minutes for 6 hours on a Zeiss Axiovert 200M using a Hamamatsu Orca camera. Images were then imported into Adobe Photoshop. Explants were outlined using the Polygonal Lasso tool to generate an approximate area for the dispersing cells. The outermost cells that originated from the explant were used to define areas. Fold changes in area were calculated by dividing all measurements by the area of each explant at the initial time point. To analyze CNC cell migration for persistence, untreated and bacEC treated explants were first photographed every 3 minutes for 10 hours to provide more comprehensive cell motility data. Time-lapse movies were then imported into Fiji and analyzed using the TrackMate plugin. The downsample log detector and simple LAP tracker were used to locate and follow cells, respectively. To avoid any effect of contact inhibition of location on persistence, only unobstructed cells were selected for analysis. Persistence was calculated by adding the total distance cells traveled and dividing by the distance traveled between the first frame and last frame of their respective paths.

## Results

### Soluble cadherin-11 ectodomain increase Akt phosphorylation *in vivo*

**Fig 1:**
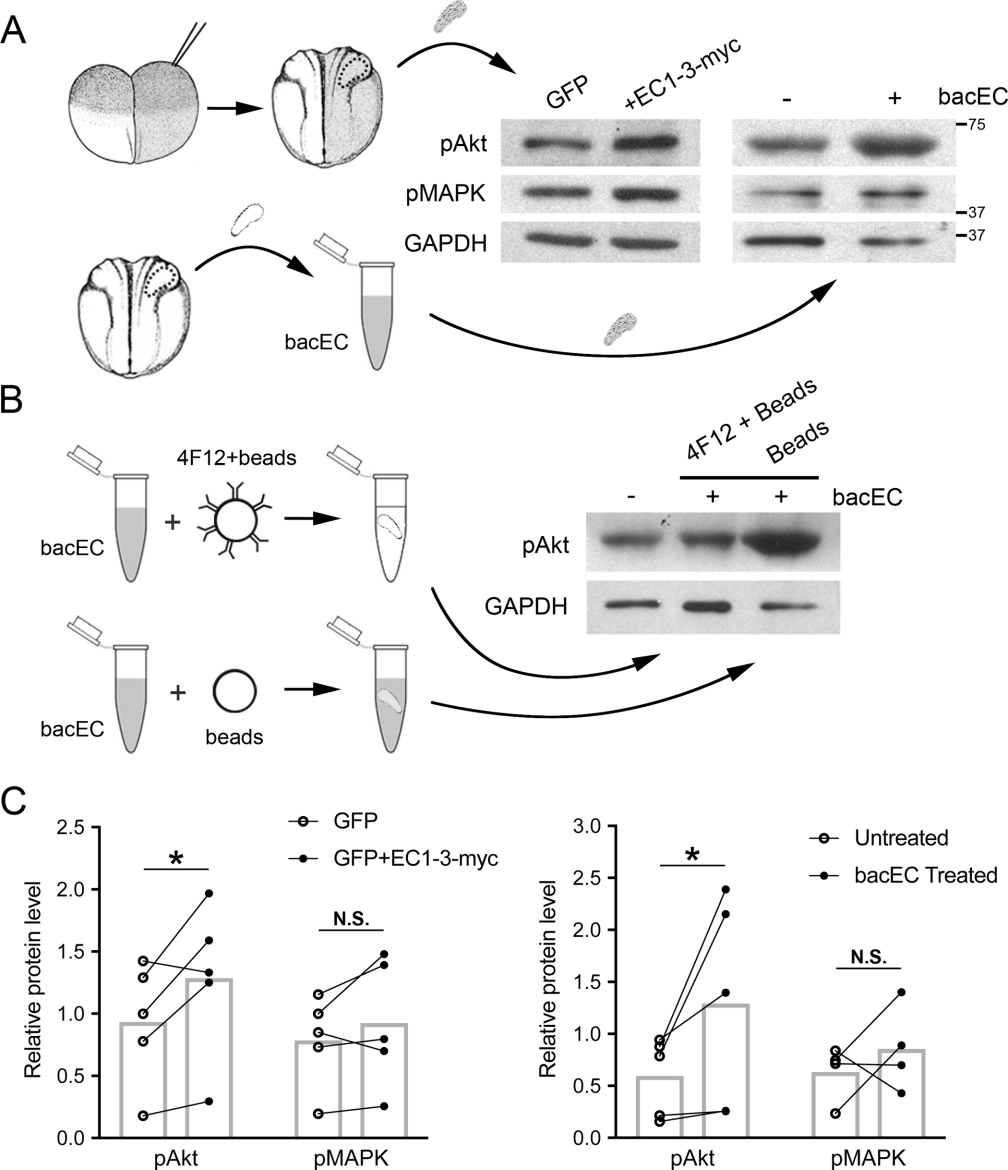
Overexpression of EC1-3-myc and treatment with recombinant EC1-3 increases Akt phosphorylation. (A, upper) Injections of 200 pg mRNA for UGP alone or together with 800 pg of EC1-3-myc mRNA were performed on single blastomeres of 2-cell *X*.*laevis* embryos. CNC expressing UGP were dissected at neurula stages, lysed and analyzed for phospho-Akt (pAkt), phospho-MAPK (pMAPK) and GAPDH via western blot. (A, lower) Non-injected CNC were dissected and treated for 5 minutes with 10 ng/mL of recombinant EC1-3 (bacEC) before being processed as above. (B) As a control, bacEC was depleted from treatment solution using a specific bacEC antibody conjugated to agarose beads and then applied to CNC. Lysates were then analyzed as above. Immunodepletion using unconjugated beads was used as a control. (C) Significant increases in phospho-Akt were observed in CNC overexpressing EC1-3-myc (N = 5, p = 0.013) and those treated with bacEC (N = 5, p = 0.021). There were not significant (N.S.) increases in the phosphorylation of MAPK in CNC overexpressing EC1-3-myc (N = 5, p = 0.09), nor those treated with bacEC (N = 5, p = 0.178). GAPDH was used as a loading control for western blots and normalizer for calculation of phospho-Akt and phospho-MAPK. Error bars are one standard deviation to the mean. One-tailed, Student’s t-tests were performed to determine statistical significance. * p<0.05. N, number of experiments.

We have previously shown that the ADAM13 metalloprotease can cleave the ectodomain of cadherin-11, and that the released fragment (EC1-3) is capable of promoting CNC migration [23]. To determine the mechanism by which EC1-3 promotes CNC migration, we first investigated whether the cadherin fragment could activate signaling pathways important for cell motility, namely the MAPK and Akt pathways [27,28,36]. We used two approaches to accomplish this. First, we overexpressed EC1-3-myc, with its signal peptide intact, along with a lineage tracer in one blastomere of 2-cell embryos and dissected CNC from the injected side once they reached neurula stage (Fig 1A). We then quantified the levels of phosphorylation of Akt and MAPK from CNC extracts by western blot (Fig 1C). Using this technique, we observed significant increases in phospho-Akt but not phospho-MAPK. To be sure that the increase in Akt activity was caused by EC1-3-myc and was not an indirect effect of long-term exposure of the embryo to the cadherin fragment, we treated CNC with a recombinant EC1-3 (bacEC; Fig 1B) and repeated the western blot analysis. Similar to the overexpression of EC1-3-myc, we found that bacEC treatment significantly increased Akt phosphorylation (Fig 1C). To confirm that bacEC and not an impurity from protein purification was responsible for the increase in phospho-Akt, we depleted bacEC from our treatment solution using an EC1-3-specific monoclonal antibody (4F12) and saw that phosphorylation of Akt did not increase (Fig 1B). To test if EC1-3 could elicit a biological response on a cellular level, we treated CNC explants with bacEC and measured various parameters of cell migration. Indeed, CNC cells migrated with a small but significantly higher persistence (S1 Fig). It is important to note that the explants were surrounded by bacEC and that its effect might be more obvious if a focused source of the cadherin-11 fragment was used. Together, these experiments demonstrate that the ectodomain of cadherin-11 can stimulate Akt activity and enhance CNC cell migration.

### Inhibition of PI3K perturbs CNC migration

**Fig 2:**
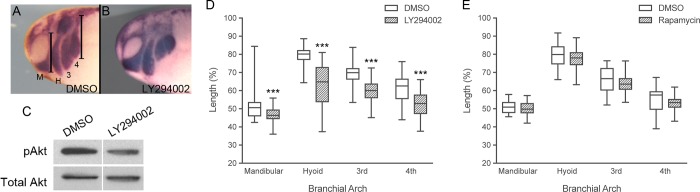
Inhibition of PI3K perturbs CNC migration. (A,B) Lateral views of tailbud stage *X*.*laevis* embryos after *in situ* hybridization with *Sox10* and *Twist* to visualize CNC positioning. Anterior is to the left, dorsal is up. Migration of CNC cells into the branchial arches is perturbed in embryos treated with 30 μM LY294002 (N = 4, n = 52). (C) Western blot of embryos treated with DMSO or 30 μM LY294002. Embryos treated from stage 17–18 to stage 25–26 with LY294002 had reduced levels of phosphorylated Akt (pAkt). One-embryo equivalents were loaded per well. (D,E) The length of CNC migration (shown in A) in each branchial arch was quantified and normalized to head size. LY294002 significantly reduced CNC migration into all branchial arches compared to DMSO controls (N = 4, n = 66). Treatment with 40 μM rapamycin (N = 4, n = 37) did not noticeably alter CNC positioning compared to DMSO controls (N = 4, n = 35, respectively). CNC cells migrated into the mandibular (M), hyoid (H), 3^rd^ and 4^th^ branchial arches. Ruler bars denote how CNC segments were measured. Error bars are one standard deviation to the mean. One-tailed, Student’s t-tests were performed to determine statistical significance. *** p<0.001. N, number of experiments; n, number of embryos.

Akt activity has recently been shown to be important for CNC migration using the inhibitor MK2206 [29]. To test if the increase in Akt phosphorylation induced by the EC1-3 was biologically relevant, we inhibited the main kinase responsible for its activation (PI3K) and observed the effects on CNC positioning using *in situ* hybridization (Fig 2). Embryos treated with the PI3K inhibitor LY294002 showed a significant reduction in branchial arch length as compared to control embryos (Fig 2A, 2B and 2D) suggesting that PI3K signaling is essential for proper CNC migration. Downstream of PI3K and Akt is the mTOR pathway, which helps regulate cell growth and proliferation [37]. Because inhibition of PI3K decreases CNC migration, we hypothesized that mTOR signaling might also be important for this process. To test this, we inhibited the mTORC1 kinase using rapamycin in the assay described above. Unlike LY294002, rapamycin had no significant effect on branchial arch length (Fig 2E). The dose of rapamycin used was shown to induce developmental defects in *Xenopus laevis* suggesting that it is capable of crossing the epidermis to affect the neural crest cells [38]. Taken together with our initial results, these data show that Akt phosphorylation is modulated by EC1-3 and that this signaling pathway is important for CNC migration.

### EC1-3 interacts with growth factor receptors

**Fig 3:**
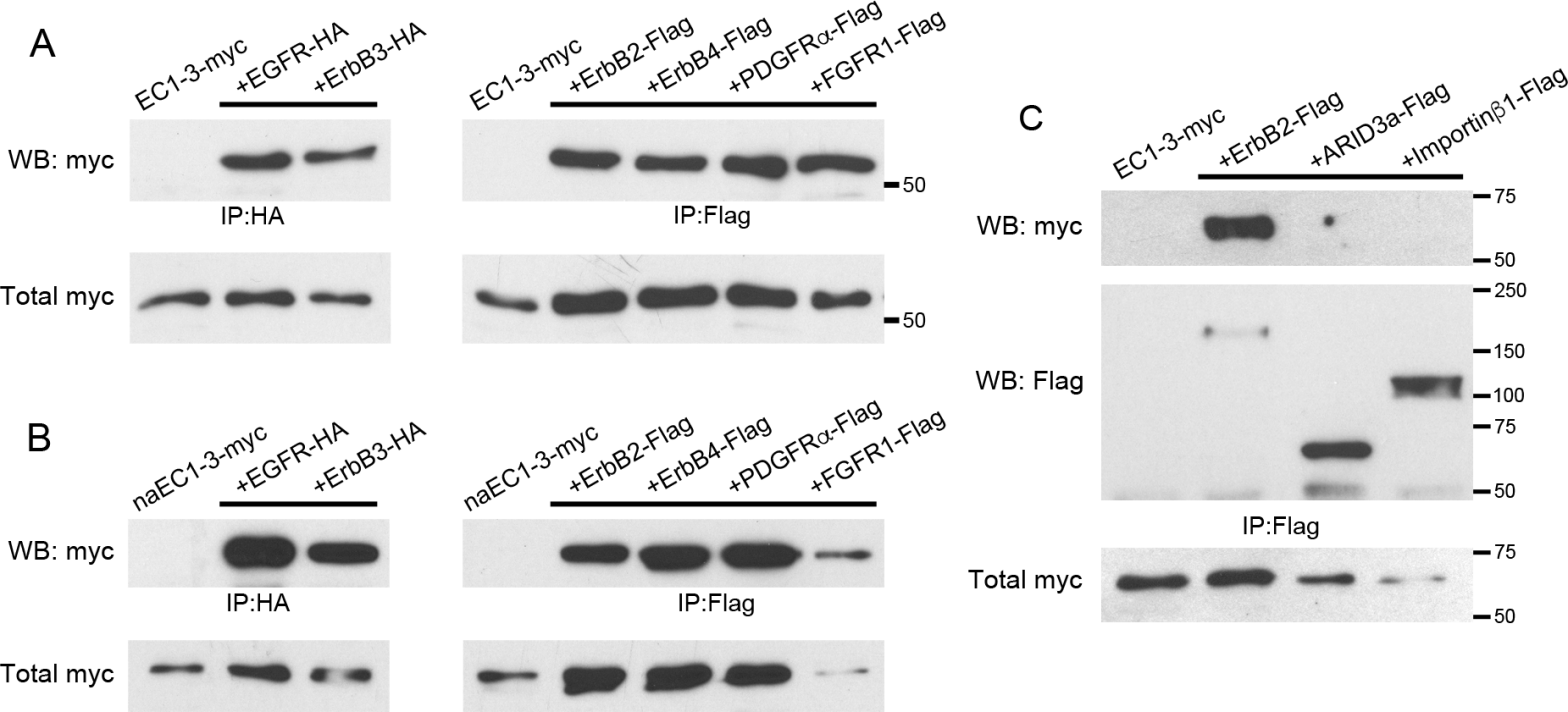
EC1-3-myc and non-adhesive EC1-3-myc interact with growth factor receptors. Western blots of EC1-3-myc (A) and non-adhesive EC1-3-myc (B; naEC1-3-myc) co-immunoprecipitations (coIPs). Receptors were immunoprecipitated using anti-HA or anti-flag antibodies. Both EC1-3 constructs co-IPed with all receptors: HA-tagged EGFR/ErbB1, HA-tagged ErbB3 and flag-tagged ErbB2, ErbB4, PDGFRα and FGFR1. (C) Western blot of EC1-3-myc coIP with cytosolic proteins ARID3a-flag and importinβ1-flag. To test if EC1-3 could interact non-specifically with proteins following extraction we tested Flag-tagged constructs overexpressed in Hek293T cells. EC1-3-myc did not associate with ARID3a-flag, nor importinβ1-flag. ErbB2-flag was used as a positive control. Results are representative of three independent experiments.

The extracellular fragment of E-cadherin has been shown to promote Akt activity by interacting with subfamilies of growth factor receptors [26–28]. Given that EC1-3 can also increase phosphorylation of Akt, we wanted to test if growth factor receptors could interact with EC1-3 to elicit this effect. To assay receptor binding with EC1-3, we transfected Hek293T cells with EC1-3-myc together with growth factor receptors naturally expressed in the CNC and performed co-immunoprecipitations (co-IPs). To our surprise, EC1-3-myc interacted with all candidate receptors: EGFR/ErbB1, ErbB2-4, FGFR1, and PDGFRα (Fig 3A). These interactions remained intact even in the presence of elevated sodium chloride (588 mM) during washes.

We have previously shown that EC1-3 can rescue CNC migration independently of the residues needed for homophilic binding [24]. To determine if EC1-3-myc can bind the growth factor receptors with its adhesive residues mutated, we repeated the experiments above with non-adhesive ectodomain (naEC1-3-myc) in place of EC1-3-myc (Fig 3B). Similar to EC1-3-myc, naEC1-3-myc was able to bind all six receptors. These data reveal a widespread and robust binding potential of EC1-3-myc to growth factor receptors. To make sure that the interaction was not happening non-specifically after protein extraction, we also performed co-IP experiments with the cytosolic proteins ARID3a-flag and importinβ1-flag (Fig 3C). EC1-3-myc did not associate with either of these proteins, suggesting that its interaction with the growth factor receptors is specific.

### Recombinant EC1-3 increases phosphorylation of Akt *in vitro*

**Fig 4:**
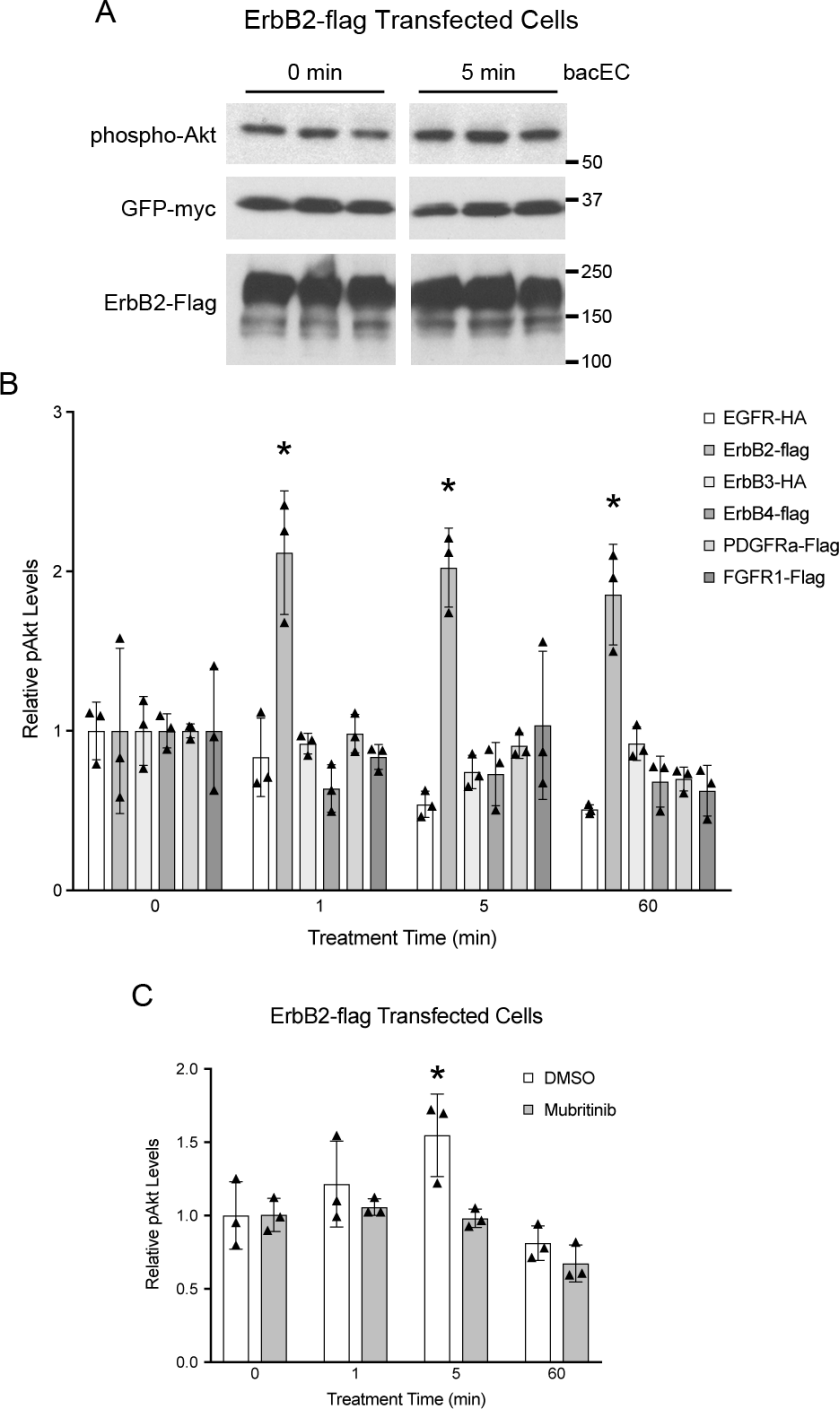
Recombinant EC1-3 (bacEC) increases Akt phosphorylation in ErbB2-transfected cells. Hek293T cells were transfected with receptor tyrosine kinases, serum-starved and treated with bacEC for indicated lengths of time. Lysates were probed for phospho-Akt (pAkt) and GFP-myc. The latter was co-transfected with receptor constructs to account for variation in transfection efficiency that could result in changes to receptor protein levels. (A) Representative western blot of lysates from ErbB2-transfected cells (in triplicate). (B,C) Quantification of pAkt from cell lysates. GFP-myc was used for normalization. (B) ErbB2-expressing cells had significantly higher pAkt levels after exposure to bacEC (p = 0.018–0.036). The p values are p = 0.02, p = 0.018 and p = 0.036 for the 1, 5, and 60 minute time points. (C) Addition of 600 nM mubritinib to ErbB2-transfected cells abrogated the effect bacEC on Akt phosphorylation. Results are representative of three independent experiments. Means were calculated from triplicates and shown as bars. Data points are shown as stars. One-tailed, Student’s t-tests were performed to determine statistical significance. * p<0.05.

To narrow down which of our candidate receptors stimulate Akt phosphorylation in response to EC1-3 binding, cells were transfected with each receptor, treated with bacEC and analyzed via western blot for Akt phosphorylation (Fig 4A). Of the six receptors tested, only ErbB2-transfected cells showed a significant increase in Akt activity in response to bacEC (Fig 4B). Increases in phospho-Akt occurred as early as one minute, and were most significant at five minutes before returning to baseline levels after sixty minutes. At their peak, phospho-Akt levels were roughly 20% higher than those of untreated cells. In contrast to ErbB2, all other receptors showed rapid decreases in Akt phosphorylation in response to bacEC and did not recover at any point during the experiment. An increase of Akt phosphorylation was also observed when EC1-3 was co-transfected with ErbB2 (S2 Fig).

To confirm that EC1-3-dependent Akt activity was mediated by ErbB2, a specific inhibitor of the receptor (mubritinib) was applied to cells before bacEC treatment. Unlike DMSO controls, cells treated with mubritinib did not increase phospho-Akt levels after bacEC treatment (Fig 4C). In fact, Akt activity decreased and remained below baselines levels similar to cells transfected with non-ErbB2 receptors.

**Fig 5:**
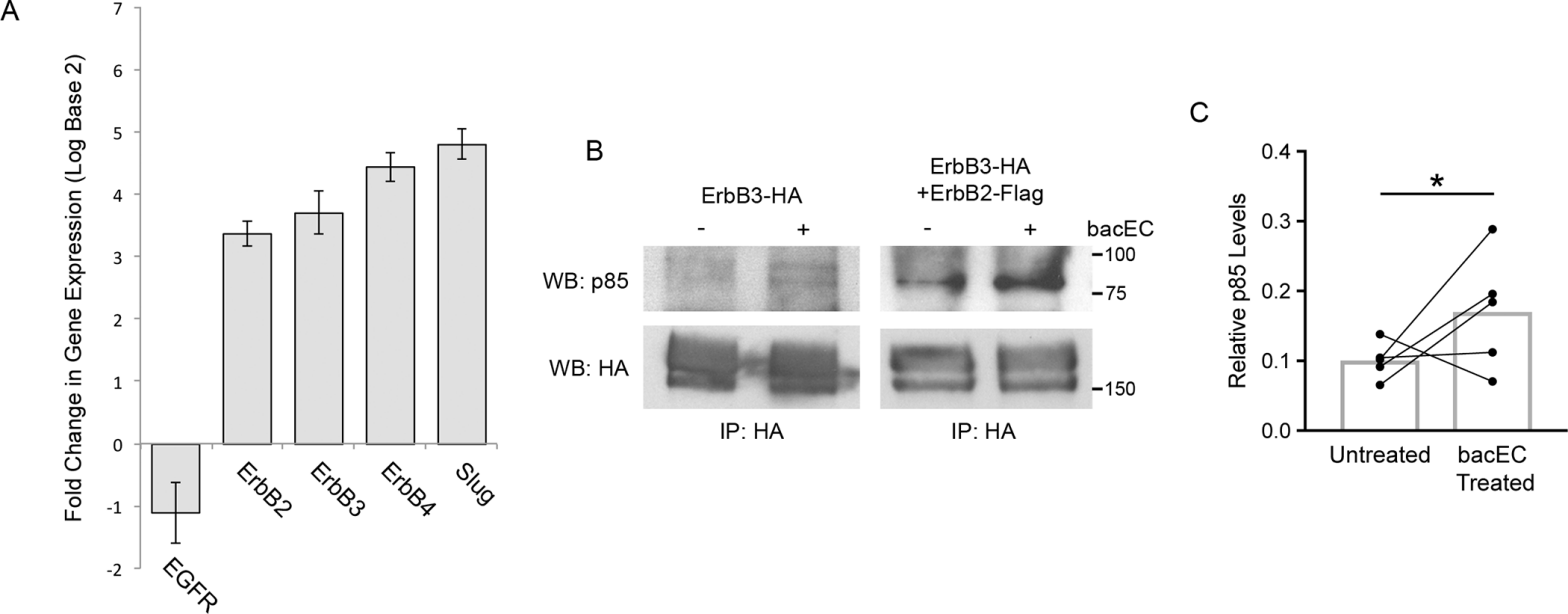
ErbB2-ErbB3 heterodimers bind PI3K p85 in the presence of recombinant EC1-3 (bacEC). (A) Quantitative real-time PCR analysis of ErbB receptors from dissected CNC and stage 16 embryos. The relative gene expression in the CNC is compared to that of whole embryos at the same stage. ErbB2, 3, and 4 are enriched in the CNC compared to whole embryos. *Slug* was used as a positive control for CNC enrichment. *GAPDH* was used for normalization. (B) Western blot of co-immunoprecipitation experiments with ErbB3-HA and p85 in the presence or absence of bacEC and ErbB2-flag. Hek293T cells were transfected with ErbB3-HA alone or together with ErbB2-flag, serum starved, and treated with 10 ng/mL bacEC for 5 minutes before being lysed for co-immunoprecipitation experiments. ErbB3-HA modestly binds p85 when transfected alone. Co-transfection of ErbB3-HA with ErbB2-flag noticeably increases this interaction. Association of p85 with ErbB3-HA is further elevated in the presence of bacEC. (C) Co-precipitation of p85 with ErbB3-HA and ErbB2-flag significantly increases (p = 0.03) in the presence of bacEC. Immunoprecipitated ErbB3-HA was used for normalization. Results are representative of three independent experiments.

Activation of Akt is preceded by a series of phosphorylation events that take place at the cell membrane. These events begin with receptor tyrosine kinases binding their cognate ligands. Afterwards, receptors dimerize and phosphorylate their cytoplasmic tails at YXXM motifs to create docking sites for the p85 subunit of PI3K [39]. While our data show that EC1-3 can bind to ErbB2 to activate Akt, the receptor is not known to homodimerize and thus requires a different ErbB receptor to activate PI3K signaling. To determine which ErbB receptor is forming a dimer with ErbB2 following EC1-3 binding, we first performed quantitative real-time PCR for the different receptors from dissected CNC and whole embryos for comparison. We found that ErbB3 and ErbB4 are enriched in the CNC along with ErbB2 (Fig 5A). We chose ErbB3 for experiments moving forward because the receptor contains more YXXM motifs than ErbB4 and is known to form a potent complex with ErbB2 upstream of PI3K/Akt signaling [40]. To determine if this complex was responsible for the signaling potential of EC1-3, we transfected ErbB2 and ErbB3 into Hek293T cells, treated the cells with bacEC and performed co-IPs for the p85 subunit of PI3K. Our results show that ErbB3 alone only modestly bound to p85, even after bacEC stimulation (Fig 5B, lanes 1 and 2). However, when co-transfected with ErbB2, ErbB3 noticeably bound to the PI3K subunit (Fig 5B, lane 3). This interaction more than doubled in bacEC-treated cells (Fig 5B, lane 4; 5C). In summary, our data show that EC1-3 can stimulate Akt phosphorylation both *in vivo* and *in vitro*, and is likely mediated by interactions between ErbB2, ErbB3 and PI3K.

### Loss of ErbB2 decreases Akt phosphorylation and CNC migration

**Fig 6:**
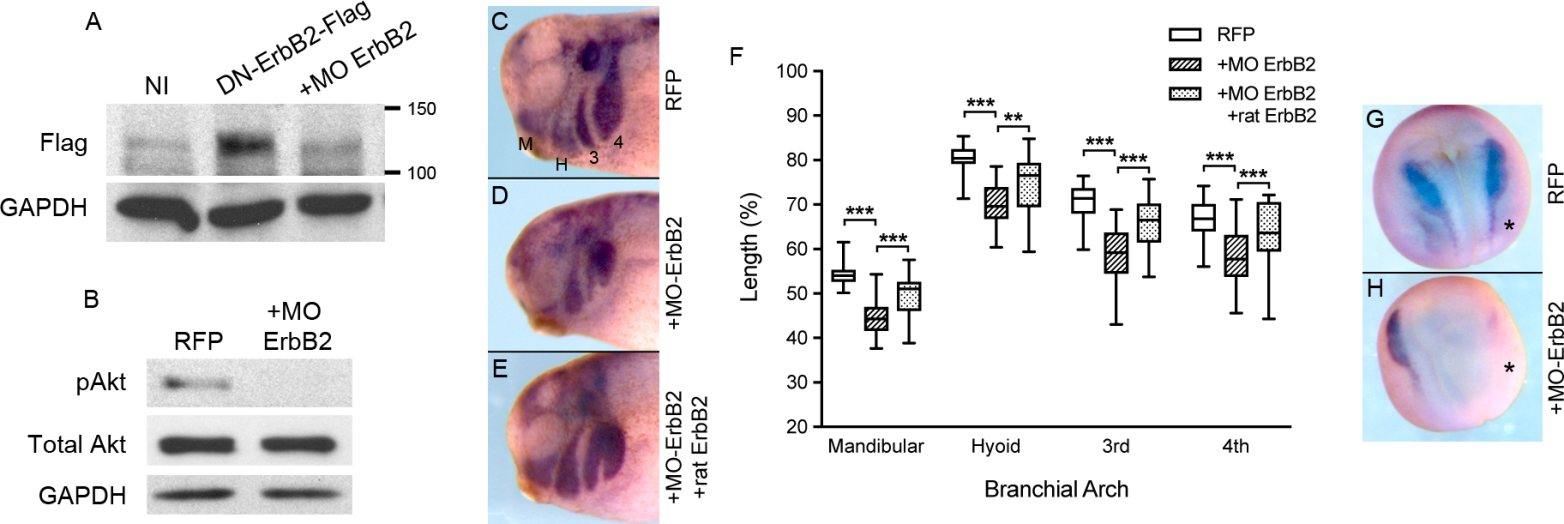
Knockdown of ErbB2 decreases phosphorylation of Akt and CNC migration. (A) Western blot of non-injected (NI) and injected embryo extracts probed for flag. Single-cell embryos were injected with 800 pg DN-ErbB2-Flag mRNA with or without 25 ng of ErbB2 morpholino (MO ErbB2). Translation of DN-ErbB2-flag was hindered in embryos co-injected with MO ErbB2. GAPDH is shown as a loading control. One-embryo equivalents are loaded per lane. (B) Western blot of CNC extracts probed for phospho-Akt, total Akt and GAPDH. Eight-cell embryos were either injected with 200 pg RFP mRNA alone (N = 4, n = 32) or with 3.1 ng MO Erb2 (N = 3, n = 43) into CNC progenitor cells. CNC cells were then dissected from embryos once they reached stage 17–18. Injection of MO ErbB2 into CNC cells precursors dramatically reduced phosphorylation of Akt without altering the levels of control proteins. Ten CNC explants are loaded in each lane. (C-E) Early tailbud stage embryos labeled for *Twist* and *Sox10* CNC markers using *in situ* hybridization. Embryos were injected as in part (B) along with a set injected with 300 pg rat ErbB2-flag and 1.6 ng MO ErbB2 (E; N = 2, n = 20). Embryos injected with RFP mRNA and MO ErbB2 showed defects in CNC migration (D) compared to RFP-injected controls (C). Migration was partially rescued by co-injecting the morpholino with rat ErbB2-flag mRNA (E). (F) CNC migration was measured in each branchial arch and normalized to head size from embryos in part (C-E). Co-injection of MO ErbB2 with RFP mRNA significantly reduced CNC migration in the mandibular (M), hyoid (H), 3^rd^ and 4^th^ branchial arches. Co-injection of MO ErbB2 with rat ErbB2 mRNA partially rescued migration. Error bars are one standard deviation to the mean. One-tailed, Student’s t-tests were performed to determine statistical significance. ** p<0.01, *** p<0.001. N, number of experiments; n, number of embryos.

Because EC1-3 binds ErbB2 to activate Akt signaling in Hek293T cells, we tested whether the receptor was important for activating Akt *in vivo* and promoting migration of CNC cells. We utilized a translation-blocking morpholino directed against ErbB2 (MO ErbB2) to answer these questions. The efficacy of MO ErbB2 was assayed by injecting it into embryos along with a flag-tagged version of the receptor (DN-ErbB2-flag). We chose this construct as it is expressed at a much higher level than the wild type receptor. Co-injection of the morpholino with receptor mRNA almost completely inhibited translation of DN-ErbB2-flag (Fig 6A). To determine if knockdown of ErbB2 in CNC cells would decrease Akt activity as we observed in mubritinib-treated cells, we injected MO ErbB2 into neural crest precursors at the 8-cell stage and dissected CNC at neurula stage for western blot analyses. Our data show that MO ErbB2 dramatically decreases phospho-Akt levels compared to control CNC (Fig 6B). To investigate whether ErbB2 was also important for CNC migration, we injected CNC precursors with MO ErbB2, along with a lineage tracer, and allowed the embryos to grow to early tailbud stages. Embryos were then collected and visualized for the CNC markers *Twist* and *Sox10* using *in situ* hybridization. Compared to CNC cells injected with RFP mRNA alone, cells co-injected with MO ErbB2 were more dorsally located (Fig 6C, 6D and 6F). This defect could be partially rescued by introducing rat ErbB2 mRNA together with MO ErbB2 into CNC precursor cells (Fig 6E and 6F). Taken together, these findings demonstrate the important role of ErbB2 in Akt phosphorylation and migration of CNC cells.

Because ErbB2 is important during gastrulation [30] and ADAM19, one of the proteases responsible for processing the EGF ligand neuregulin1β [41], is important for CNC induction [42], we also tested if loss of ErbB2 interferes with induction of the CNC. To test this, we performed *in situ* hybridization on injected embryos prior to the onset of CNC migration (stage 18). Our results show that knockdown of ErbB2 in CNC precursors did not appear to affect gastrulation but significantly decreases the expression of the CNC markers *Sox10* and *Twist* (Fig 6G and 6H).

**Fig 7:**
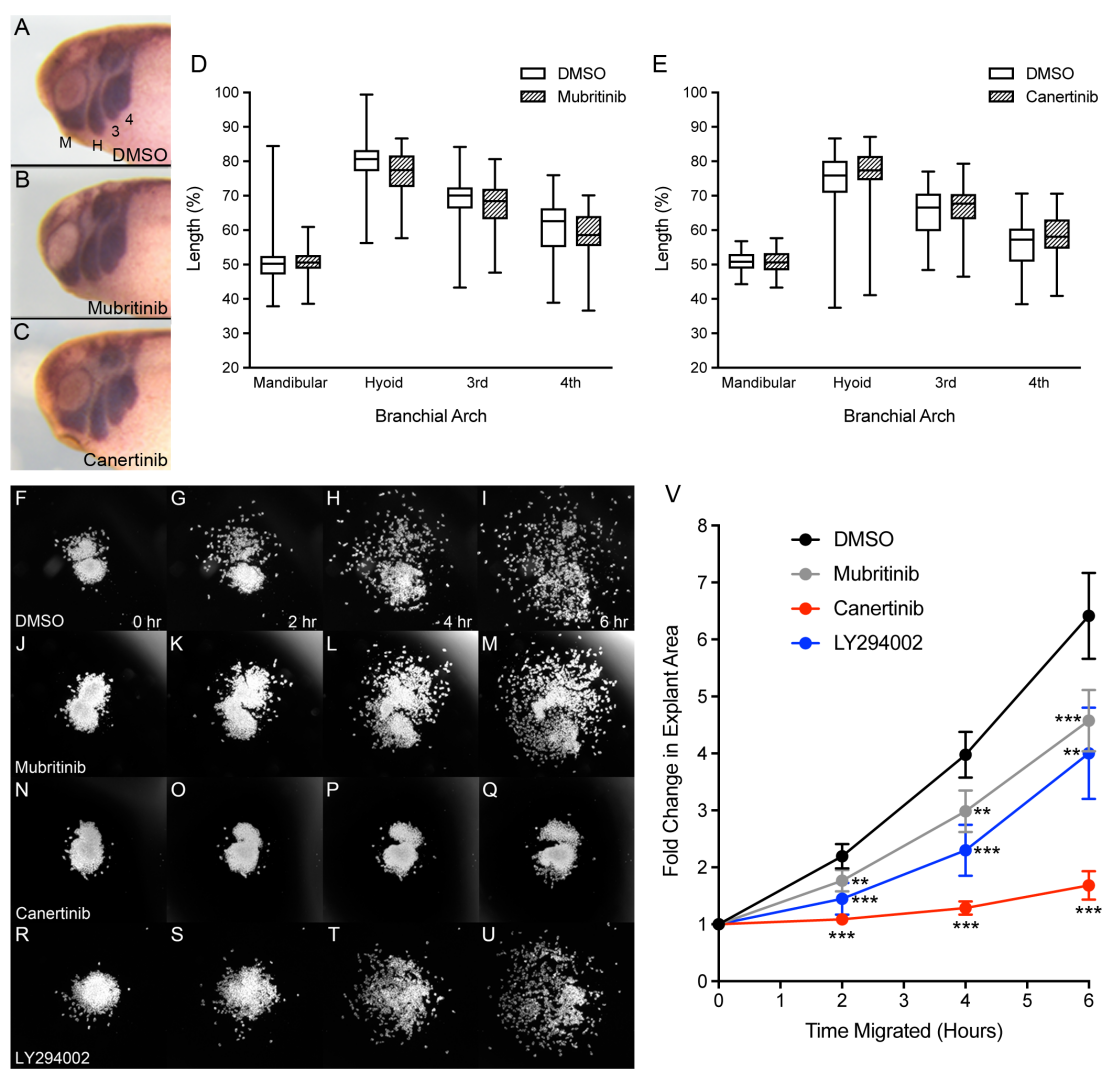
Mubritinib and canertinib perturb CNC migration *ex vivo*. (A-C) Lateral views of tailbud stage *X*.*laevis* embryos after *in situ* hybridization with *Sox10* and *Twist* to visualize CNC positioning. Anterior is to the left, dorsal is up. Embryos treated with 40 μM of the ErbB2 inhibitor, mubritinib (N = 4, n = 72), or 25 μM of the pan-ErbB inhibitor, canertinib (N = 4, n = 68), show no difference in CNC migration compared to DMSO controls (N = 6, n = 100; N = 4, n = 69, respectively). (D-E) The distance of migration within each branchial arch was measured and normalized to the head size. CNC migration as observed in the mandibular (M), hyoid (H), 3^rd^ and 4^th^ branchial arches. (F-U) Time-lapse images of CNC explants migrating on fibronectin substrate and treated with 0.5% DMSO (N = 8, n = 24), 6 μM mubritinib (N = 4, n = 12), 10 μM canertinib (N = 4, n = 12), and 20 μM LY294002 (N = 3, n = 9) for indicated lengths of time. (V) Fold change in explant surface area over time. Areas were normalized to measurements calculated at initial time points (t = 0). All inhibitors significantly decreased CNC migration *ex vivo*. Error bars represent the 95% confidence interval. One-tailed, Student’s t-tests were performed to determine statistical significance. ** p<0.01, ***p<0.001. N, number of experiments; n, number of embryos or explants.

Given that knockdown of ADAM13 does not affect Slug, Sox10 nor Twist gene expression [43], we expect that the role of ErbB2 in neural crest induction is independent of the cadherin-11 extracellular fragment. To test the contribution of ErbB2 in CNC migration without affecting CNC induction, we treated embryos with a specific inhibitor of ErbB2 (mubritinib) and a pan-ErbB inhibitor (canertinib) during CNC migration. Embryos were grown and processed for *in situ* hybridization as before. To our surprise, the inhibitors had little effect on CNC migration *in vivo* (Fig 7A–7E). To test if the inhibitors could successfully traverse the ectoderm and several layers of CNC cells, we tested whether mubritinib and canertinib could affect phospho-Akt levels in neurula-stage embryos. Consistent with the lack of phenotypes observed above, neither inhibitor was able to decrease phospho-Akt levels compared to embryos treated with DMSO (S3 Fig).

Because mubritinib can inhibit Akt phosphorylation by blocking ErbB2 in Hek293T cells (Fig 4C) and *Xenopus* XTC cells (S4 Fig), we hypothesize that mubritinib and canertinib are unable to decrease Akt activity, and therefore affect CNC migration, because they cannot efficiently penetrate deep embryonic tissues. To overcome this obstacle, CNC explants were dissected, place on fibronectin with inhibitors, and monitored for six hours. After two hours of treatment, mubritinib and canertinib had already decreased CNC migration compared to DMSO-treated CNC (Fig 7K and 7O). This trend continued for the duration of the experiment as significant decreases were observed at the four and six-hour marks for both inhibitors (Fig 7L, 7M, 7P and 7Q). We were also able to distinguish differences between the inhibitors. While inhibition of ErbB2 (mubritinib) reduced cell persistence (Fig 7V; gray), inhibition of multiple ErbB receptors (canertinib) nearly halted CNC migration altogether (Fig 7V; red). To determine whether inhibition of PI3K signaling had a similar effect to mubritinib or canertinib, we treated CNC explants with LY294002 and again performed time-lapse microscopy (Fig 7R–7U). In agreement with our *in vivo* data, inhibition of PI3K significantly reduced CNC migration *ex vivo* (Fig 7V, blue). These data show that ErbB2 and PI3K play important roles in CNC migration.

## Discussion

### What is the role of cadherin ectodomain shedding?

Cadherin ectodomain shedding has been shown to regulate multiple different biological processes. For example, shedding of E-cadherin and VE-cadherin regulates epithelial cell extrusion and endothelial cell adhesion, respectively [44,45]. Cleavage of N-cadherin can control neurite outgrowth as well as neural stem cell quiescence [46,47]. Proteolysis of cadherins has also been linked to pro-migratory behaviors but predominantly in the context of cancer progression. The other more physiological instance is during neural crest cell development.

Cleavage of cadherins ectodomains is an important step for proper development of neural crest (NC) cells. In avian models, ADAM10 cleaves N-cadherin and cadherin-6B to promote delamination and epithelial-mesenchymal transition of NC cells, respectively [5,6]. The initial extracellular cleavage ultimately triggers the release of the cadherin cytoplasmic tail from the membrane, allowing it to regulate gene expression [5,20]. The function of the shed extracellular fragment has not been investigated in this context. In the case of cranial neural crest (CNC) cells, however, cadherin-11 cleavage by ADAM13 produces a soluble extracellular N-terminal fragment (EC1-3) that is capable of rescuing migration in cells overexpressing cadherin-11 as well as cells lacking ADAM13 [23]. The ability of EC1-3 to promote CNC migration does not require its homophilic binding site. However, this site is necessary for EC1-3 to inhibit contact inhibition of locomotion [24]. This suggests that EC1-3 could play two independent roles during CNC migration: one that regulates cell motility independent of its homophilic domain, the other acting as a competitive inhibitor of integral cadherin-11 during contact inhibition of locomotion.

### How do cadherin ectodomains modulate cell motility?

Recent studies of the shed extracellular domain of E-cadherin (sEcad) showed that it could signal through Akt to stimulate cell migration [27,28]. Using LY294002 to inhibit PI3K/Akt signaling, Brouxhon and colleagues showed that the pro-migratory effect of sEcad on cancer cells could be abrogated *in vitro*. When we treated *Xenopus laevis* embryos with the same inhibitor, we observed a modest but significant reduction in CNC migration *in vivo*. Interestingly, we did not observe any effect on CNC migration by inhibiting the mTOR pathway (rapamycin), which is commonly found downstream of Akt signaling. This suggests that Akt signaling relies on other downstream pathways to mediate cell migration in this context. For example, Akt might regulate the cytoskeleton via palladin or girdin [48,49]. In the presence of a chemoattractant gradient, *Dictyostelium* cells accumulate PI3K along their leading edge where it helps maintain cell polarity for cell migration [50,51]. Because cadherin-11 is cleaved by ADAM13 throughout CNC migration [23], it is possible that a gradient of EC1-3 mediates an accumulation of PI3K, and thus Akt, along the cell membrane. This could ultimately promote CNC migration by helping the cells establish polarity and cytoskeletal projections. In support of this hypothesis, a recent study of early CNC migration showed that Akt activity is required to maintain expression of N-cadherin, which is critical for promoting contact inhibition of locomotion of the CNC [10,29]. Thus, local shedding of cadherin-11 could provide a small increase of Akt activation and N-cadherin expression at the onset or during CNC migration.

### ErbB2 is a receptor for the shed ectodomain of cadherin-11

Association of sEcad with ErbB receptors has been shown to activate Akt signaling and thereby promote migration of cancer cells [26–28]. Pharmacological inhibition of one or more of these receptors significantly reduces the pro-migratory effect of sEcad [26–28]. Despite the differences between type I (E-cadherin) and type II (cadherin-11) classical cadherins, we show that the shed extracellular domain of cadherin-11 (EC1-3) is also capable of binding all four ErbB receptors. We also show that inhibition of ErbB2 or multiple ErbB receptors significantly reduces cell migration of CNC explants. This finding, combined with the ability of EC1-3 to increase Akt phosphorylation, suggests that EC1-3 binds to ErbB2 at the surface of CNC to stimulate Akt and promote cell migration. What makes this interaction fascinating is that ligand binding is not necessary for ErbB2 to adopt an active conformation prior to dimerization [52]. It is conceivable that EC1-3 stabilizes ErbB2 interactions with dimerization partners as has been shown for sEcad and ErbB2-ErbB3 heterodimers [25]. Our results showing increased binding of the p85 subunit of PI3K with ErbB3 in the presence of bacEC and ErbB2 supports this mechanism of action for EC1-3. It is also worth noting that the protocadherin PCNS is also cleaved by ADAM13 and that the shed extracellular domain can also promote CNC migration [53], suggesting that interactions between cadherin ectodomains and growth factor receptors is a widely conserved phenomenon. Identification of the binding site of cadherins on growth factor receptors could shed the light on this apparent lack of specificity.

ErbB receptors respond to multiple EGF ligands during development. ADAM metalloproteases can mediate these signaling pathways independent of cadherin cleavage. For example, ADAM10 and ADAM17 can release EGF ligands from the plasma membrane. ADAM19 has been shown to cleave the HB-EGF ligand and play a critical role in cardiac neural crest cell development in mouse [54,55]. Interestingly, the knockdown of ADAM19 in *X*.*laevis* embryos also reduces the level of phospho-Akt and neural crest markers similar to what we have observed here with MO ErbB2 [42]. Thus, attributing the various roles of ADAM and EGF signaling during CNC migration will not be a simple task.

### Does EC1-3 use other growth factor receptors to regulate cell migration?

Loss of cleavage of cadherin-11 results in a much more dramatic inhibition of CNC migration than the inhibition of PI3K or Akt suggesting that EC1-3 does not function exclusively via ErbB2/Akt signaling [23,24,29]. Similarly, the loss of ErbB2 produces a wide range of phenotypes (e.g. gastrulation delay) that are likely independent of cadherin-11 processing. Therefore, the wide range of receptors that EC1-3 can bind may be relevant to its function *in vivo*. Cranial neural crest cells rely on a variety of receptor-ligand interactions for proper guidance during migration [56]. Two of these receptors, FGFR1 and PDGFRα, have also been known to bind full-length cadherins and impart a new function on the receptor [36,57]. While it is clear that only ErbB2 generated an increase in phospho-Akt, it is possible that the other receptors we tested responded to EC1-3 through different signaling cascades. This is true even for ErbB receptors, as inhibition of ErbB2 alone does not affect *ex vivo* cell migration to the extent of canertinib, the pan-ErbB receptor inhibitor. This suggests that the signaling pathways initiated by non-ErbB2 dimers are also important for CNC migration. Some of these pathways may not rely on Akt signaling but instead involve phospholipase-C gamma 1 (PLCγ1), Janus kinases (Jak) and Signal Transducer and Activator of Transcription (STATs), which can be found downstream of PDGF and FGF signaling [58–61].

EC1-3 may also coordinate a response from multiple receptors to regulate CNC cell motility. Experiments identifying the level of phosphorylation of these receptors in response to EC1-3 will be required to answer this question. The difficulty of testing the response to EC1-3 lies in the fact that inhibition of each of these receptors can have profound effects on embryogenesis [30,62,63]. Without the knowledge of the precise binding site of EC1-3 on each receptor, it is impossible to selectively inhibit EC1-3 binding without affecting all of the other receptor functions.

Inhibition of ErbB2 or multiple ErbBs using mubritinib and canertinib, respectively, significantly reduced CNC migration *ex vivo*. When we analyzed the treated explants for phospho-Akt, we observed no reduction in the activated protein level. This is in contrast to the knockdown of ErbB2, which showed a drastic reduction in phospho-Akt levels in the CNC (Fig 6B). The evidence of phenotypic alteration by the drug, while not affecting the level of phospho-Akt, suggests that ErbB receptors may depend on a different secondary messenger during CNC migration. Alternatively, it is possible that other receptors are the primary controllers of Akt phosphorylation. For example, the PDGFRα receptor expressed in *X*.*laevis* CNC controls Akt phosphorylation in gain of function experiments [29]. However, decreases in phospho-Akt were not shown in loss of function experiments suggesting that multiple pathways may regulate Akt activity. Given the effectiveness of the ErbB inhibitors in decreasing phospho-Akt level in Hek293T cells, it is clear that cellular responses to ErbB2/EC1-3 binding might differ depending on the cell type. Our identification of ErbB2 as a validated partner of EC1-3 will better enable us to uncover the specific binding site of the cadherin-11 ectodomain and elucidate its mechanism of action with other receptors in future studies.

## Conclusion

**Fig 8:**
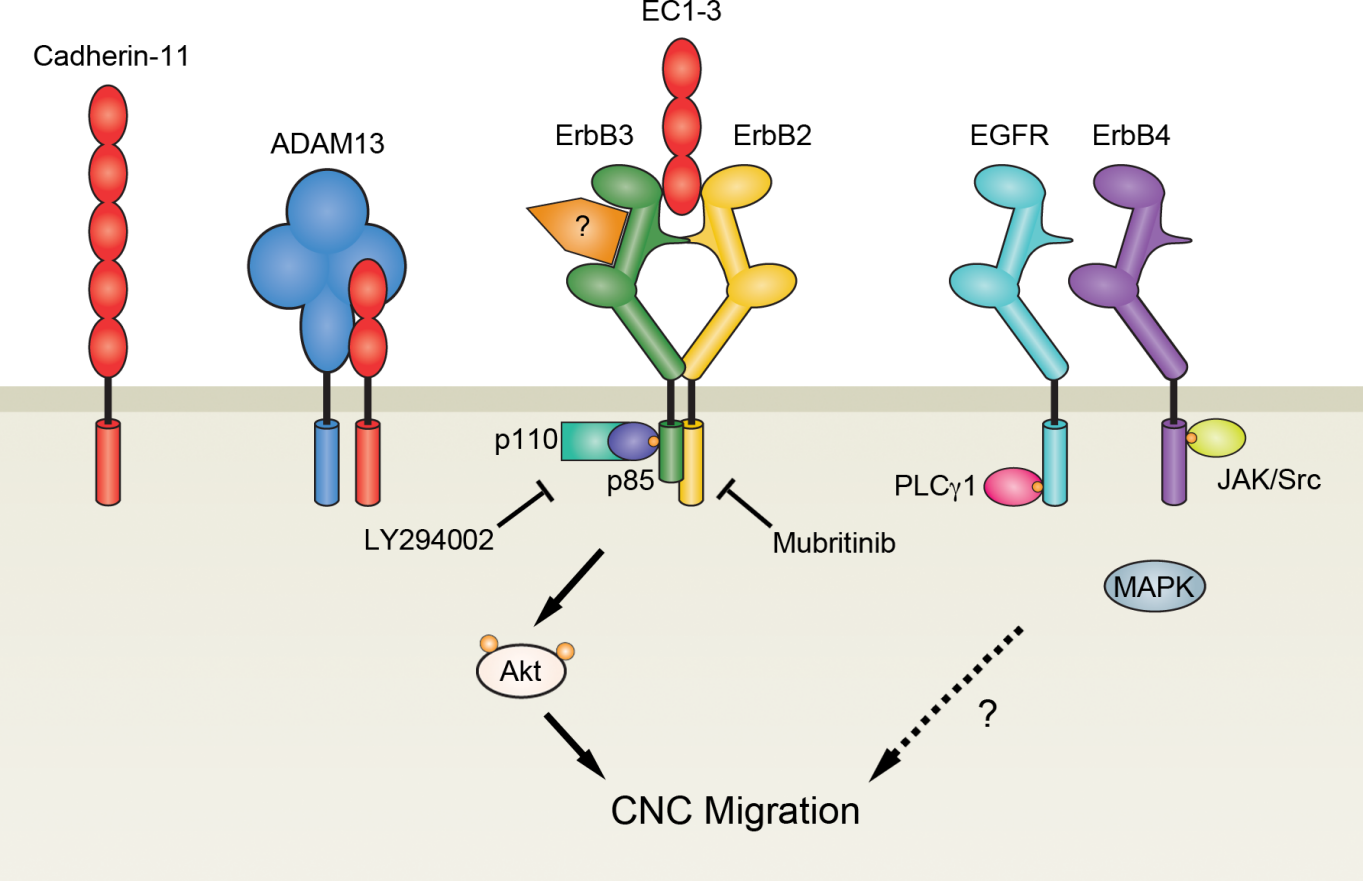
EC1-3 stimulates Akt and CNC migration. During migration of cranial neural crest (CNC) cells, ADAM13 (blue) cleaves the extracellular domain of cadherin-11 (red) at the cell surface to release a soluble fragment (EC1-3) capable of promoting CNC migration. EC1-3 can induce cell migration by binding to ErbB receptors: EGFR/ErbB1 (teal), ErbB2 (yellow), ErbB3 (green) and ErbB4 (purple). In particular, binding of EC1-3 with ErbB2-ErbB3 dimers increases docking of the regulatory (p85) subunits of PI3K. Recruitment of PI3K to the cell membrane ultimately leads to the phosphorylation of Akt, which is important for CNC migration. Because inhibition of ErbB2 is less detrimental to cell migration than inhibition of all ErbB receptors, it is possible that EC1-3 utilizes Akt-independent pathways downstream of ErbB dimers that do not include ErbB2. Whether EC1-3 signaling first requires receptors to bind their cognate ligand still needs to be investigated.

Cleavage of cadherin ectodomains has been observed as a necessary step to promote neural crest migration [5,6,23]. While studies have shown how type I classical cadherin fragments can mediate this process in cancer cells [27,28], our data show how the shed fragment of the type II classical cadherin, cadherin-11, can promote this process in CNC cells by interacting with ErbB2 to increase Akt activity (Fig 8). Because ADAM13, the metalloprotease responsible for cadherin-11 cleavage, has other substrates in the cadherin superfamily, it will be worth investigating if the shed ectodomains of these cadherins can modify cell behavior similar to EC1-3 [24,53].

## Supporting information

S1 Fig Recombinant EC1-3 increases the persistence of migrating CNC cells. (A) Persistence of cell migration was calculated by adding the distances cells traveled between time points (d) and dividing it by their net displacement (D). (B,C) Quantification of persistence and velocity for untreated and recombinant EC1-3 (bacEC) treated CNC cells. CNC explants were dissected from neurula stage embryos and incubated on fibronectin substrate for at least one hour before being treated with 10 ng/mL bacEC and monitored using time-lapse microscopy. Analysis of cell movement revealed that CNC cells treated with bacEC (N = 3, n = 6, c = 103) have a significantly higher persistence (p = 0.013) than untreated cells (N = 3, n = 6, c = 99). No statistical difference was observed in CNC cell velocity. Scatter plot shows median and interquartile range. One-tailed, Student’s t-tests were performed to determine statistical significance. * p<0.05. N, number of experiments; n, number of explants; c, number of cells.
(TIF)

S2 Fig Co-expression of EC1-3 with ErbB2 increases Akt phosphorylation *in vitro*. Hek293T cells were transfected with a nuclear-localized RFP (nucCherry), EC1-3-myc, ErbB2, or a combination of EC1-3-myc and ErbB2. Cells were then serum starved for at least 18 hours and lysed for western blotting. GAPDH was used as a loading control. Results are representative of three independent experiments.
(TIF)

S3 Fig Mubritinib and canertinib do not inhibit Akt phosphorylation in embryos. Western blot of embryos treated with 30 μM LY294002, 40 μM mubritinib, 25 μM canertinib or DMSO from stage 18 to stage 25–26. Of the inhibitors, only LY294002 was able to decrease phosphorylation of Akt (pAkt) compared to DMSO controls. GAPDH was used as a loading control. One-embryo equivalents were loaded per lane.
(TIF)

S4 Fig Mubritinib inhibits Akt phosphorylation in *Xenopus* XTC cells. Western blot of XTC cells transfected with *X*.*laevis* ErbB2 and GFP-myc, serum starved for 18 hours, and treated with either DMSO or 600 nM mubritinib for one hour. Mubritinib dramatically decreased phosphorylation in one of two Akt isoforms (pAkt) compared to DMSO-treated controls. GFP-myc was co-transfected with ErbB2 to account for variation in transfection efficiency that could result in changes to receptor protein levels.
(TIF)
