# Supplementary figures and images for: The ectodomain of cadherin-11 binds to erbB2 and stimulates Akt phosphorylation to promote cranial neural crest cell migration
Source: PLoS One. 2017 Nov 30;12(11):e0188963. doi: 10.1371/journal.pone.0188963 (PMC5708760; doi:10.1371/journal.pone.0188963)

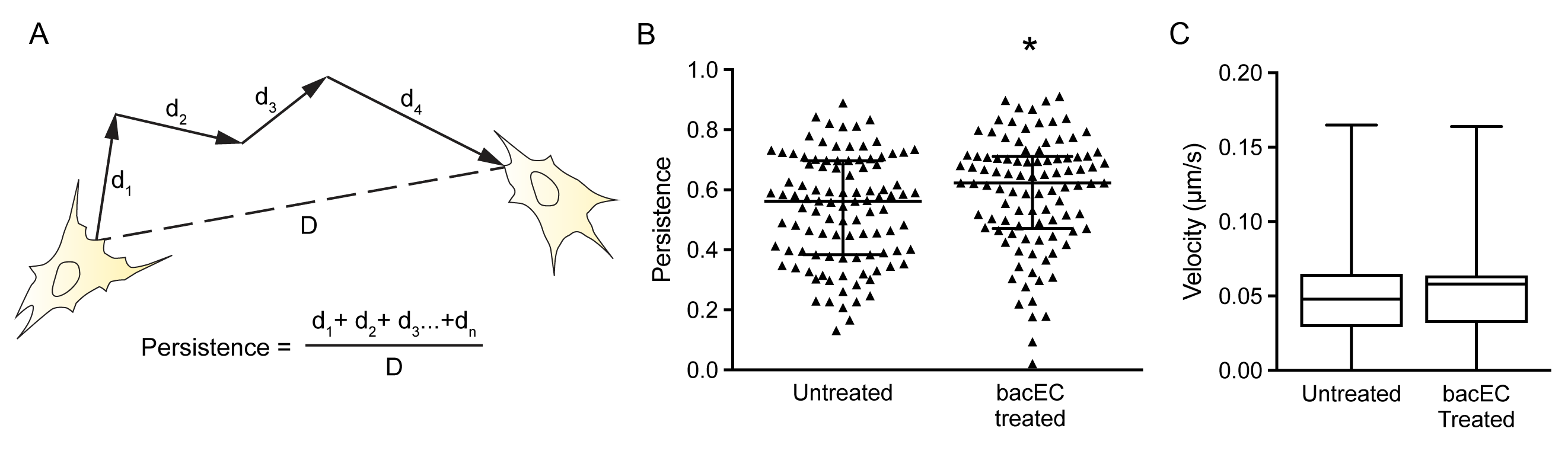

Supplement: S1 Fig — (A) Persistence of cell migration was calculated by adding the distances cells traveled between time points (d) and dividing it by their net displacement (D). (B,C) Quantification of persistence and velocity for untreated and recombinant EC1-3 (bacEC) treated CNC cells. CNC explants were dissected from neurula stage embryos and incubated on fibronectin substrate for at least one hour before being treated with 10 ng/mL bacEC and monitored using time-lapse microscopy. Analysis of cell movement revealed that CNC cells treated with bacEC (N = 3, n = 6, c = 103) have a significantly higher persistence (p = 0.013) than untreated cells (N = 3, n = 6, c = 99). No statistical difference was observed in CNC cell velocity. Scatter plot shows median and interquartile range. One-tailed, Student’s t-tests were performed to determine statistical significance. * p<0.05. N, number of experiments; n, number of explants; c, number of cells. (TIF) [file pone.0188963.s001.tif]

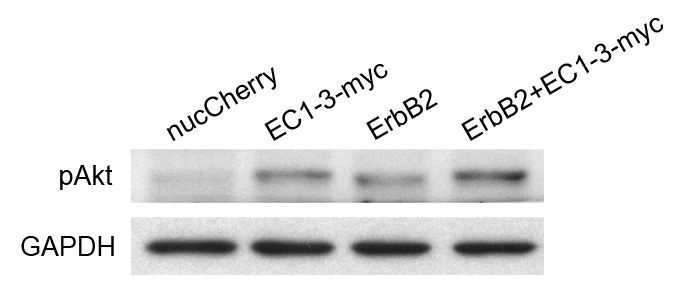

Supplement: S2 Fig — Hek293T cells were transfected with a nuclear-localized RFP (nucCherry), EC1-3-myc, ErbB2, or a combination of EC1-3-myc and ErbB2. Cells were then serum starved for at least 18 hours and lysed for western blotting. GAPDH was used as a loading control. Results are representative of three independent experiments. (TIF) [file pone.0188963.s002.tif]

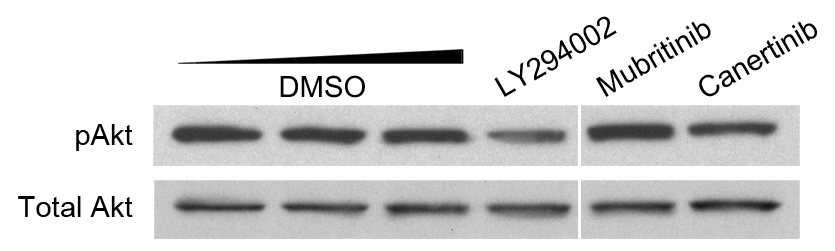

Supplement: S3 Fig — Western blot of embryos treated with 30 μM LY294002, 40 μM mubritinib, 25 μM canertinib or DMSO from stage 18 to stage 25–26. Of the inhibitors, only LY294002 was able to decrease phosphorylation of Akt (pAkt) compared to DMSO controls. GAPDH was used as a loading control. One-embryo equivalents were loaded per lane. (TIF) [file pone.0188963.s003.tif]

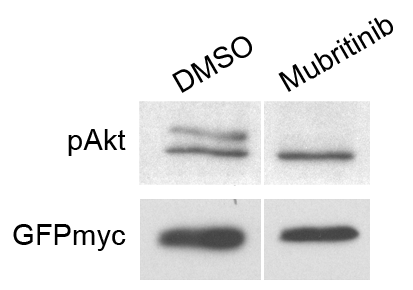

Supplement: S4 Fig — Western blot of XTC cells transfected with X.laevis ErbB2 and GFP-myc, serum starved for 18 hours, and treated with either DMSO or 600 nM mubritinib for one hour. Mubritinib dramatically decreased phosphorylation in one of two Akt isoforms (pAkt) compared to DMSO-treated controls. GFP-myc was co-transfected with ErbB2 to account for variation in transfection efficiency that could result in changes to receptor protein levels. (TIF) [file pone.0188963.s004.tif]
